# Smoking and the Genetic Contribution to Alcohol-Dependence Risk

**Published:** 2000

**Authors:** Pamela A. F. Madden, Kathleen K. Bucholz, Nicholas G. Martin, Andrew C. Heath

**Affiliations:** Pamela A. F. Madden, Ph.D., is an assistant professor of psychology; Kathleen K. Bucholz, Ph.D., is a research associate professor of epidemiology; and Andrew C. Heath, D.Phil., is the Spencer T. Olin Professor of Psychiatry in the Department of Psychiatry. They all teach at the Washington University School of Medicine in St. Louis, Missouri, and are associated with the Missouri Alcoholism Center. Nicholas G. Martin, Ph.D., is a professor of genetics in the Division of Epidemiology at Queensland Institute of Medical Research, Brisbane, Australia

**Keywords:** smoking, AOD (alcohol or other drug) dependence potential, genetic correlation analysis, genetic theory of AODU (AOD use, abuse, and dependence), twin study, risk factors, family AODU history, alcohol cue, chronic AODE (effects of AOD use, abuse, and dependence)

## Abstract

Genes influence a person’s risk of becoming a smoker as well as the risk of alcohol dependence. Because substantially higher rates of smoking are observed in alcoholics than in control groups, uncovering the mechanisms underlying this association may have important implications for both treatment and prevention. Data analyses from the 1981 Australian twin panel cohort confirm a positive genetic correlation between regular smoking and the risk of alcohol dependence that remains significant, even when sociodemographic and personality variables as well as histories of other psychopathologies are taken into account. Acute or chronic effects of smoking on subjective responses to alcohol may play a role in this association.

Genes are known to play a role in determining a person’s risk for becoming a regular smoker as well as his or her risk of becoming dependent on alcohol. In determining the contributions of genetic and environmental factors to the development of alcohol and tobacco problems, researchers frequently use two important tools—twin and adoption studies. Investigations using twins yield important information about the relative contribution of genetic and environmental factors to the development of a disease, such as alcoholism. Identical (i.e., monozygotic) twins share 100 percent of their genes, whereas fraternal (i.e., dizygotic) twins share, on average, 50 percent of their genes. Adoption studies typically compare the outcomes of adoptees who have biological parents with alcohol problems who grow up in various adoptive environments with the outcomes of adoptees without such family backgrounds who are raised in similar environments.

As reviewed elsewhere (e.g., Heath 1995; Heath et al. 1997*b*) and supported by more recent twin studies (e.g., True et al. 1996; Heath et al. 1997*a*; Prescott et al. 1999), twin and adoption studies provide remarkably consistent evidence for an important genetic contribution to alcohol-dependence risk, accounting for as much as 40 to 60 percent of the total variation in risk in people of European ancestry. Alcoholism (variously defined) in adoptees is predicted by alcoholism in the adoptees’ biological parents rather than in their adoptive parents (e.g., Goodwin et al. 1973; Cloninger et al. 1981; Cadoret et al. 1994). In addition, rates of alcohol dependence are much higher in monozygotic than in dizygotic co-twins of alcohol-dependent twins. Accumulating evidence from twin studies also indicates an important genetic contribution to the risk of nicotine dependence (e.g., True et al. 1999; Kendler et al. 1999). Major twin studies from Scandinavia, Britain, Australia, and America provide consistent evidence for a powerful genetic influence on the risk of becoming a regular smoker—where regular smoking typically has been defined as having smoked 100 or more cigarettes during one’s lifetime (e.g., Madden et al. 1999; for reviews, see Heath and Madden 1995; Heath et al. 1998)—with estimates of heritability comparable to those observed for alcohol dependence. Heritability is the proportion of the total variation in risk of becoming a regular smoker that is accounted for by genetic factors.

As discussed elsewhere in this issue, researchers have established that rates of smoking are substantially elevated in alcoholics and have observed this finding both in clinical and in general community samples (Istvan and Matarazzo 1984; Glassman et al. 1990). We understand relatively little, however, about how the strong association between smoking and problems with alcohol arises. Twin analyses suggest an important overlap or correlation in the genetic factors that contribute to the risk for nicotine and alcohol dependence (True et al. 1999) as well as between measures of smoking and alcohol use (Prescott and Kendler 1995) and heavy drinking (e.g., Swan et al. 1997). However, reporting correlations—even genetic correlations—tells us little about why a genetic relationship apparently exists between smoking and alcohol dependence. A number of different possibilities need to be considered, including the following:

- *Shared risk factors.* Personality, sociodemographic variables, and history of a psychiatric disorder increase both risk of alcohol dependence and risk of smoking.
- *Direct causal influence of smoking (or vice versa).* Smoking per se increases the risk that a person will develop problems with alcohol or, less plausibly (because of the typically earlier onset of regular smoking than of alcohol dependence), alcohol dependence increases the risk that a person will become a regular smoker.
- *Shared genetic vulnerability.* Such vulnerability may arise, for example, because of alcohol’s effects on important molecules (i.e., nicotinic receptors) involved in brain cell (i.e., neuron) communication.

These different hypotheses may have different implications for attempts to prevent or treat alcohol problems. Data from the 1981 cohort of the Australian twin panel (Kendler et al. 1986; Martin et al. 1985; Heath et al. 1994, 1997*a*) allow us to explore the issue of shared genetic risk factors between alcohol dependence and smoking.

## Smoking and Alcohol Dependence in the Australian Twin Panel

The Australian twin panel is a volunteer national twin sample maintained by the Australian National Health and Medical Research Council. Between 1980 and 1982, a questionnaire survey of adult twins registered with the panel produced replies from both members of approximately 3,800 twin pairs. Researchers conducted a followup telephone diagnostic interview survey between 1992 and 1994. The baseline questionnaire included questions about the respondents’ smoking histories. From the duration of smoking reported in these data, respondents who had smoked only once or twice apparently classified themselves as “never smokers.” Therefore, a positive response on the smoking section of the survey indicated a history of regular smoking rather than a history of experimentation with cigarettes. By combining these data with information about lifetime prevalence of alcohol dependence according to the American Psychiatric Association’s *Diagnostic and Statistical Manual, Third Edition, Revised* (DSM–III–R), as reported in the interview survey, the usual finding can be replicated, indicating a strong association between smoking and alcohol dependence.

As shown in table 1, without adjusting for covariates (e.g., sociodemographic and personality variables or a history of psychopathology), women with histories of regular smoking were five to six times more likely to report histories of alcohol dependence in the 1992–1994 survey compared with women who reported not smoking. In addition, men with histories of regular smoking were approximately two times more likely to report alcohol dependence compared with men who reported not smoking. Thus, the association between smoking and alcohol dependence was significantly stronger in women (the odds ratio, a measure of the strength of the association between two binary variables, was 5.9) than in men (an odds ratio of 3.0).

As shown in table 2, the more alcohol-dependence symptoms they reported (whether current or former), the more likely people were to report a history of regular smoking. Among smokers, however, no significant relationship existed between the number of cigarettes typically consumed per day and the risk of alcohol dependence in either gender (not shown).

The genetic overlap between smoking and a history of alcohol dependence can be assessed many different ways. One approach is to use a simple statistical model (i.e., logistic regression model), in which a respondent’s alcohol-dependence history is predicted as it relates to gender, twin-pair zygosity type (i.e., monozygotic or dizygotic), co-twin’s smoking history (i.e., current or former smoker vs. nonsmoker), and interactions between these variables.

If a significant genetic correlation exists between smoking and alcohol dependence, smoking in one twin should correlate more highly in monozygotic than in dizygotic pairs, with alcohol dependence experienced by the co-twin. The two-way interaction between whether twins are monozygotic or dizygotic (i.e., twin-pair zygosity) and whether one twin reports a history of smoking provides a direct statistical test for this genetic correlation (Heath et al. 1998).

As shown in table 3, without adjusting for covariates, a significant two-way interaction is observed.^1^ When covariates are included—for example, extraversion, neuroticism, tough-mindedness, and social nonconformity; frequency of church attendance and religious affiliation; educational level; and history of major depression and conduct disorder (see Heath et al. 1997*b* for further details)—the effect of the two-way interaction is reduced but remains significant.^2^ In other words, the influence of sociodemographic, personality, and psychiatric risk factors on both the risk of alcohol dependence and the risk of regular smoking is not sufficient to explain the observed genetic association between smoking and alcohol dependence**.**

## Smoking and Reactions to Alcohol

In an important series of investigations, Schuckit and colleagues have shown that when compared with control participants, young adult males with family histories of alcoholism exhibit diminished reactions to a standardized dose of alcohol (i.e., a challenge test). Those reactions included the subjects’ subjective ratings of alcohol effects and their degree of body-sway compared with a baseline measurement taken before testing (e.g., Schuckit 1980, 1985). Although this work has been criticized on a number of methodological grounds (e.g., Newlin and Thompson 1990), Schuckit has completed a followup study showing that a low response to alcohol’s effects when originally tested predicts an increased risk of alcohol dependence at followup (Schuckit and Smith 1996). As we discuss below, these findings may provide one important clue as to how the association between smoking and alcohol dependence may arise. Schuckit has hypothesized that one subgroup of alcoholics may inherit a diminished reactivity to alcohol and because they experience less intoxication, less body-sway, or other similar effects after consuming a specified amount of alcohol, they are able to drink more heavily before becoming impaired and, thus, are more likely to progress to more harmful levels of alcohol use.

Shortly before the 1981 questionnaire survey of the Australian twin panel, researchers initiated an alcohol challenge study in which 206 young adult male and female twin pairs were tested with a challenge dose of alcohol (Martin et al. 1985). Schuckit hypothesized that the alcohol reactivity measures that he was using were, at least in part, genetically determined. The Australian Alcohol Challenge Twin Study (AACTS) confirmed significant evidence for genetic effects on body-sway increase and subjective intoxication rating after drinking alcohol, with a combined measure of alcohol reactivity (based on both body-sway and intoxication) showing 60 percent heritability (Heath et al. 1999).

The Australian study differed in important respects from the studies conducted by Schuckit. First, the Australian study did not attempt to recruit people with positive family histories of alcoholism. Second, the study did not use the exclusionary criteria considered standard in contemporary research (e.g., people with histories of alcohol problems at baseline were not excluded). As a consequence of this (and probably unique for this type of research), people with and without family histories of alcoholism and people who were light or heavy drinkers, or who had problems with alcohol, participated in this study in approximately the same proportions as would be observed for the general population of Australians of this age group (Heath et al. 1999). In other words, participants (at least the men) broadly represented all twins of their age group from the Australian twin panel, showing few significant differences from those twins who did not participate. Twins who participated in the original 1981 Australian twin study who could be located also were included in the 1992 diagnostic interview followup survey. Similar to the research conducted by Schuckit, even when drinking history and alcohol problems reported at the time of the original challenge study were controlled for, people with histories of alcohol dependence at interview followup had significantly lower scores on the alcohol reactivity measure at baseline.

Furthermore, people, at least men, who reported not having histories of alcohol dependence but who were at higher-than-average genetic risk, because they were monozygotic co-twins of alcohol-dependent participants, showed the same diminished responses to alcohol in the challenge study. And nondependent people at intermediate genetic risk who had dizygotic co-twins with histories of alcohol dependence had scores that fell between the two former high-risk groups and the control group (Heath et al. 1999). For reasons not yet understood, comparable associations were not observed in women.

Consistent with Schuckit’s research, findings from the AACTS suggest that men who inherit a low reactivity to alcohol have an increased risk of alcohol dependence. Currently, we do not know which genes are involved in this reduced reactivity to alcohol. Genetic variations (i.e., polymorphisms) in a key enzyme involved in alcohol metabolism (i.e., alcohol dehydrogenase) at two genes called ADH2 and ADH3 did not predict differences in alcohol reactivity, even though the ADH2*2 gene variant (i.e., allele) predicted diminished alcohol-dependence risk in men from this sample (Whitfield et al. 1998). However, analyses of the relationship between smoking history and alcohol challenge performance produced important further insights. Men and women who were current smokers at the time of the alcohol challenge study rated themselves as significantly less intoxicated than did nonsmokers or former smokers despite receiving the same amount of alcohol (Madden et al. 1995). This relationship persisted in women, even when a history of heavy drinking was controlled for, and also was observed in men at the second post-alcohol assessment. Furthermore, in women, researchers found a highly significant genetic correlation between smoking status and self-report of intoxication after the alcohol challenge (Madden et al. 1997). In men, the familial association between smoking status and post-alcohol intoxication appeared to be largely attributable to similar alcohol-relevant experiences between twins. Because of the small sample sizes, however, we cannot exclude the possibility that a genetic correlation existed in the men.

Thus, either acute or chronic cross-tolerance effects may exist between smoking and alcohol or some similar interaction that leads to diminished response to alcohol in those who smoke. Alternatively, common genetic mechanisms may lead to the increased probability that a person will become a regular smoker and to reduced propensity for intoxication after a given dose of alcohol. These effects appear to be much stronger in women than in men, consistent with the much stronger association between smoking and alcohol problems in women noted in table 1.

Several limitations of the original AACTS make it difficult to determine whether the study results show a direct interaction between smoking and alcohol use or common underlying genetic mechanisms. Participants in the study were allowed to smoke if they so wished, but their smoking during the experimental session was not documented. Consequently, we were unable to separate the effects of smoking histories from smoking during the challenge session. Furthermore, because the study used a quasi-random sampling scheme and because monozygotic twin pairs exhibit a high concordance for regular smoking, few smoking discordant pairs (i.e., where only one twin smoked) participated in the protocol. Researchers are currently addressing these issues more directly in ongoing challenge studies using separate and joint administration of nicotine and alcohol to adult nonsmokers and smokers.

## Conclusions

For many years both clinicians and researchers have acknowledged the strong comorbidity between smoking and alcohol dependence. This issue cannot be explained simply in terms of shared individual risk factors. Genetic approaches are likely to play an important role in helping to document the biological mechanisms underlying this association. The experimental finding that smokers appear to experience less intoxication after a challenge dose of alcohol than do nonsmokers, combined with evidence that low reactivity to alcohol predicts increased long-term risk of alcohol dependence, suggests that further experimental studies of nicotine-alcohol interactions using genetically informative approaches should be a research priority.

## Figures and Tables

**Table 1:** Association Between Alcohol Dependence* and Respondents’ Smoking History

Smoking History	*n*	Female Respondents (*n* = 3,840)			*n*	Male Respondents (*n* = 2,006)		
	
		Alcohol Dependent (%)	Odds Ratio	95% Confidence Interval		Alcohol Dependent (%)	Odds Ratio	95% Confidence Interval
Ex-smoker	1,554	11.5	5.9	4.3–8.2	998	30.7	3.0	2.6–3.6
Nonsmoker	2,286	2.1	1.0	—	1,008	16.9	1.0	—

* The terms “alcohol dependence” and “alcohol dependent” are defined according to the American Psychiatric Association’s *Diagnostic and Statistical Manual, Third Edition, Revised*.

Odds Ratio = measure of the strength of the association between two binary variables.

Confidence interval = range of the strength of association between variables, consistent with the observed data.

**Table 2:** Association Between Number of Alcohol-Dependence Symptoms* and History of Regular Smoking

	Number of Alcohol-Dependence Symptoms							

Regular Smokers	0	1	2	3	4	5	6	7–9
Women (%)	32.0	54.0	64.0	71.8	87.5	77.3	71.4	94.1
Men (%)	36.6	50.6	56.3	59.2	61.4	68.4	75.9	83.3

* Alcohol-dependence symptoms are based on the American Psychiatric Association’s *Diagnostic and Statistical Manual, Third Edition, Revised.*

**Table 3:** Association Between Alcohol Dependence and Co-Twins’ Smoking Status With and Without Adjustment for Covariates*

	Unadjusted		Adjusted for Covariates	
	
	Odds Ratio	95% Confidence Interval	Odds Ratio	95% Confidence Interval
**Gender (Male = 1**)	5.59	4.45–7.02	5.01	3.87–6.48
**Zygosity (MZ = 1)**	0.44	0.25–0.77	0.56	0.31–1.01
**Co-Twin Smoking Status**	1.20 NS	0.68–2.11	1.01 NS	0.55–1.86
**Zygosity x Co-Twin**				
**Smoking Status**	3.13	1.46–6.71	2.52	1.13–5.65

* Covariates included personality variables, sociodemographic variables, and histories of conduct disorder and major depression. See Heath and colleagues (1997*a*) for full details of covariates.

MZ = monozygotic or identical twins; NS = nonsmoker.

Odds Ratio = measure of the strength of the association between two binary variables.

Confidence interval = range of the strength of association between variables, consistent with the observed data.

## Footnotes

1 Odds ratio 3.13, a 95-percent confidence interval of 1.46–6.71.

2 Odds ratio 2.52; a 95-percent confidence interval of 1.13–5.65.
